# AMY-tree: an algorithm to use whole genome SNP calling for Y chromosomal phylogenetic applications

**DOI:** 10.1186/1471-2164-14-101

**Published:** 2013-02-13

**Authors:** Anneleen Van Geystelen, Ronny Decorte, Maarten HD Larmuseau

**Affiliations:** 1 UZ Leuven, Laboratory of Forensic Genetics and Molecular Archaeology, Leuven, Belgium; 2 Department of Biology, KU Leuven, Laboratory of Entomology, Leuven, Belgium; 3 Department of Imaging & Pathology, KU Leuven, Forensic Medicine, Kapucijnenvoer 33, Leuven, B–3000, Belgium; 4 Department of Biology, KU Leuven, Laboratory of Biodiversity and Evolutionary Genomics, Leuven, Belgium

**Keywords:** Haploid marker, Phylogeny, Next-generation sequencing, SNP calling, Y-SNP mutations, Y chromosome haplogroups

## Abstract

**Background:**

Due to the rapid progress of next-generation sequencing (NGS) facilities, an explosion of human whole genome data will become available in the coming years. These data can be used to optimize and to increase the resolution of the phylogenetic Y chromosomal tree. Moreover, the exponential growth of known Y chromosomal lineages will require an automatic determination of the phylogenetic position of an individual based on whole genome SNP calling data and an up to date Y chromosomal tree.

**Results:**

We present an automated approach, ‘AMY-tree’, which is able to determine the phylogenetic position of a Y chromosome using a whole genome SNP profile, independently from the NGS platform and SNP calling program, whereby mistakes in the SNP calling or phylogenetic Y chromosomal tree are taken into account. Moreover, AMY-tree indicates ambiguities within the present phylogenetic tree and points out new Y-SNPs which may be phylogenetically relevant. The AMY-tree software package was validated successfully on 118 whole genome SNP profiles of 109 males with different origins. Moreover, support was found for an unknown recurrent mutation, wrong reported mutation conversions and a large amount of new interesting Y-SNPs.

**Conclusions:**

Therefore, AMY-tree is a useful tool to determine the Y lineage of a sample based on SNP calling, to identify Y-SNPs with yet unknown phylogenetic position and to optimize the Y chromosomal phylogenetic tree in the future. AMY-tree will not add lineages to the existing phylogenetic tree of the Y-chromosome but it is the first step to analyse whole genome SNP profiles in a phylogenetic framework.

## Background

A large part of the human Y chromosome, named the nonrecombining Y chromosomal portion or NRY, is strictly paternally inherited. Due to the absence of recombination during meiosis for this Y chromosomal portion, it is possible to define the hierarchic descent order of all human NRY variants and to infer the order and time of their descent in the phylogenetic tree, as the coalescence theory describes [1]. The phylogenetic framework of the Y chromosome has important applications in a wide range of fields including evolutionary anthropology and population history [1-4], genetic genealogy [5,6], medical genetics [7,8] and forensic sciences [9,10].

The last published version of the full phylogenetic tree with all human NRY lineages, named haplogroups, was published by [11]. This tree includes 316 Y chromosomal lineages and is based on approximately 600 described binary markers, mostly Y chromosomal Single Nucleotide Polymorphisms (Y-SNPs) but also Y chromosomal indels. However, the number of (sub-)haplogroups that may be interesting for several applications is expected to be much higher. This expectation is based on the thousands of unknown Y-SNPs which are observed in recent whole genome studies like the Irish genome project [12], on the presence of an abundant number of polytomies in the phylogenetic tree of several haplogroups [11], and on the many recent publications with new Y chromosomal lineages [13,14]. Also, numerous described sub-haplogroups are still clearly paraphyletic within network analyses based on Y chromosomal Short Tandem Repeats (Y-STRs), for example sub-haplogroup G-P303* and J-M410* [15,16]). Based on these Y-STR networks, it is clear that it is still not possible to distinct several phylogenetic groups using Y-SNPs which may be relevant for several disciplines and applications of the Y chromosomal tree.

It is expected that an explosion of Y chromosomal data will become available in the near future considering the increasing number of next-generation sequencing (NGS) studies. DNA sequencing efficiency has increased by approximately 100,000-fold in the last decade; next-generation sequencing machines may now resequence the entire human genome in only a few days [17]. This whole genome resequencing combined with highly efficient analysis software is being used to uncover large numbers of SNPs – SNP calling – and structural variants – SV calling – in the human genome according to a reference genome [18]. Therefore, it is expected that an exponential number of new Y-SNPs and a growing number of (sub-)haplogroups will be described in the forthcoming years which will resolve the Y chromosomal tree much further as the tree will be much more branched. This will also lead to an increased phylogenetic resolution for many Y chromosome studies. However, scientists working with the Y chromosomal tree will need to deal with several issues in the post-genomic era:

1) *Automated determination of the Y chromosomal lineage*. Currently, to determine the Y chromosomal haplogroup of a sample for a broad population study, all required binary markers are still genotyped individually or in limited multiplexes by RFLP, SNaPshot, TaqMan or even by direct sequencing [7,19,20]. However, the Y chromosomal phylogenetic tree is expected to grow substantially to such an extent that it will be impossible and inefficient to genotype and analyse each Y-SNP individually (or in multiplexes) in order to define the Y chromosomal lineage of a sample. Therefore, it is required to automate the determination of the (sub-)haplogroup based on NGS data in a time-efficient manner.

2) *Detection of new Y-SNPs*. Currently, new Y-SNPs are mainly found by sequencing a small portion of the Y chromosome for a limited number of individuals of a certain haplogroup, introducing ascertainment bias [13,21,22]. Therefore, it is necessary to detect Y-SNPs with yet unknown phylogenetic position (yupp-SNPs) based on whole genome data. Moreover, to integrate all those not yet reported Y-SNPs easily into the phylogenetic framework it is required that they are identified time-efficiently and checked in all available whole genome Y-SNP profiles.

3) *Control of the currently used Y chromosomal tree*. There are two possible ways to update the currently used Y chromosomal phylogenetic tree: first, making *Tabula rasa* of the current phylogenetic tree and obtaining a new phylogeny based on only the high quality whole sequenced Y-chromosomes, or secondly, controlling the current phylogeny and including new validated Y-SNPs based on whole sequenced Y-chromosomes. The *tabula rasa* option has several drawbacks mainly since there are not yet enough qualitative samples from all regions and of all sub-haplogroups and since continuity between present and future Y-chromosomal studies is required. The currently used Y-chromosomal tree seems to be very robust and the backbone seems to be solid [23]. Therefore, expanding this tree is currently the best solution. The current Y chromosomal phylogenetic trees need to be in accordance with the available whole genome data. If whole genome data is correct and this data is not in accordance with the phylogeny, it means that the Y chromosomal phylogeny needs to be adapted. It is presumed that the most recent phylogenetic tree may include some mistakes due to the current huge time effort to type each Y-SNP separately in all reported Y chromosomal lineages. Therefore, it is expected that recurrent mutations will occur in particular lineages with strong effect on several Y chromosomal analyses [24]. Moreover, it is not always clear what the correct mutation conversion is for several Y-SNPs, in other words which allele of the Y-SNP is ancestral and which one is mutant due to contradiction in different papers or resources.

4) *Updating of the Y chromosomal tree*. The state of the art Y chromosomal phylogeny is difficult to follow up based on the literature, even for scientists working exclusively on this chromosome. Moreover, it will be extremely complicated when more SNPs will be reported in the post-genomic period. Often it is the case that a publication shows new Y-SNPs within a particular haplogroup, however, it does not mention the phylogenetic position of previous reported Y-SNPs in the revised Y chromosomal tree which makes it very difficult to integrate the new with old data. For example, a new set of Y-SNPs reshaped recently the phylogeny of haplogroup G in [16]. however, some Y-SNPs which were described in a previous revision of haplogroup G in [22] were not included in the [16] phylogeny. As such it is very difficult to maintain an updated version of the full Y chromosomal phylogeny and these problems will increase in the future.

Here, we present an automated approach as a first solution to these four challenges of using whole genome SNP calling data in future Y chromosomal research. This methodology has been implemented in a software package, called ‘AMY-tree’, and validated on data available from several already published whole genome SNP calling projects. Therefore, AMY-tree is the first step to analyse whole genome SNP profiles in the existing phylogenetic framework.

## Methods

### AMY-tree software

**Figure 1:**
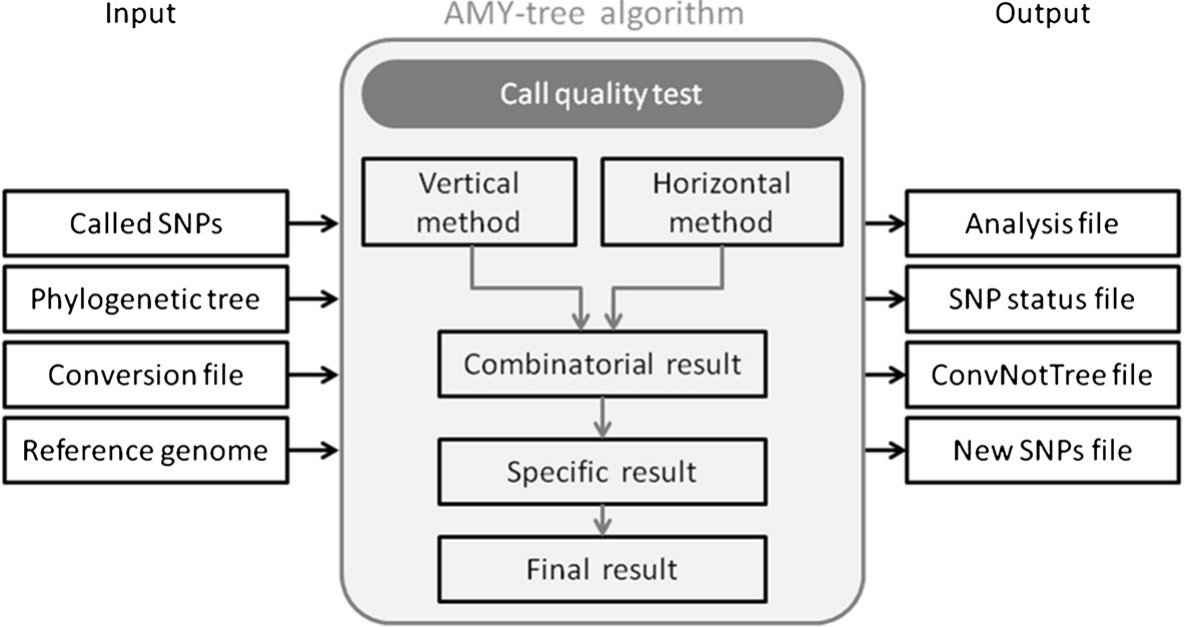
**Workflow of the full AMY-tree software package.** The input of AMY-tree are the called Y-SNPs for an individual under study, a Y chromosomal phylogenetic tree, mutation conversion data of all known Y-SNPs and the reference genome. In the algorithm, AMY-tree checks first the quality of the called Y-SNPs via the ‘Call quality test’. Then, vertical and horizontal methods are applied to assign the sub-haplogroup of the sample and their results are combined to get the combinatorial result. In order to remove more false positive results, the most specific result is retained and returned as final result. The output of AMY-tree is the analysis file with the assignment of the sub-haplogroup for the sample and three files which make it possible to check, correct and expand the phylogenetic tree.

The AMY-tree software package is written in Perl; its workflow is shown in Figure 1. Based on the whole genome SNP calling profile, AMY-tree will determine the (sub-)haplogroup of a sample in a time-efficient manner. Therefore, it needs all the data of the called SNPs, a Y chromosomal phylogenetic tree, a Y-SNP mutation conversion file and the reference genome that was used to call the SNPs. Besides the result with the final (sub-)haplogroup, the software also generates specific data files which make it possible to check, correct and expand the phylogenetic tree.

#### Input

The SNP calling data file that will be used as one of the input files for AMY-tree contains all Y-SNPs of a single individual which are called by comparing the whole genome sequences with the reference genome. Since SV calling is still difficult and inefficient in whole genome sequencing applications [25], we rejected indels from the analysis to maintain the required quality for AMY-tree. For example, it is hard to be sure if a mutation on M17 within R1a1 is present in a sample based on only NGS data as the indel mutation conversion is 4G->3G. The format of the SNP calling file is a simple tab separated values format such that called SNPs of most prediction programs and sequencing platforms can be used. We created software, the so-called ‘WHY conversion tool’, which can convert the formats and which is described later on.

Another input file is the Y-SNP conversion file containing all the essential data of the Y-SNPs for which mutation conversion is scientifically reported. The mutation conversion of a Y-SNP is always reported by its ancestral and mutation allelic states, e.g. M173: A->C with A the ancestral and C the mutant allele state of M173. As a single (new reported) SNP has often several names when the SNP is detected in different labs independently, the synonyms of the SNP are also given, e.g. M173 = P241 = Page29. Besides the name, mutation conversion and possible synonyms of the SNP, the position of the SNP on the Y chromosome (on both NCBI build 36/hg18 and GRCh37/hg19) is also present in this file which is based on the information given in [11], all published papers with newly reported Y-SNPs, the ISOGG-website (http://www.isogg.com) and the Y Chromosome Browser of Family Tree DNA (ymap.ftdna.com). See Additional file 1: Table S1 for the most recent SNP conversion list May 2012.

The Y chromosomal phylogenetic tree file contains the Y chromosomal phylogenetic data. It will not be possible in the future to draw all Y chromosomal lineages in a clear graphical way once there will be an exponentially growth of Y-SNP data. Therefore, we choose here for a simple tab separated values format that is easy to understand and created by the user. We have made a Y chromosomal phylogenetic tree file based on the [11] tree and another one based on all the published updates of the [11] tree (see further).

AMY-tree also requires a fasta file of the reference genome that was used to call the SNPs because the ancestral or mutant state of all SNPs in the phylogenetic tree needs to be determined. This is most of the times NCBI build 36/hg18 or GRCh37/hg19.

#### Algorithm

**Figure 2:**
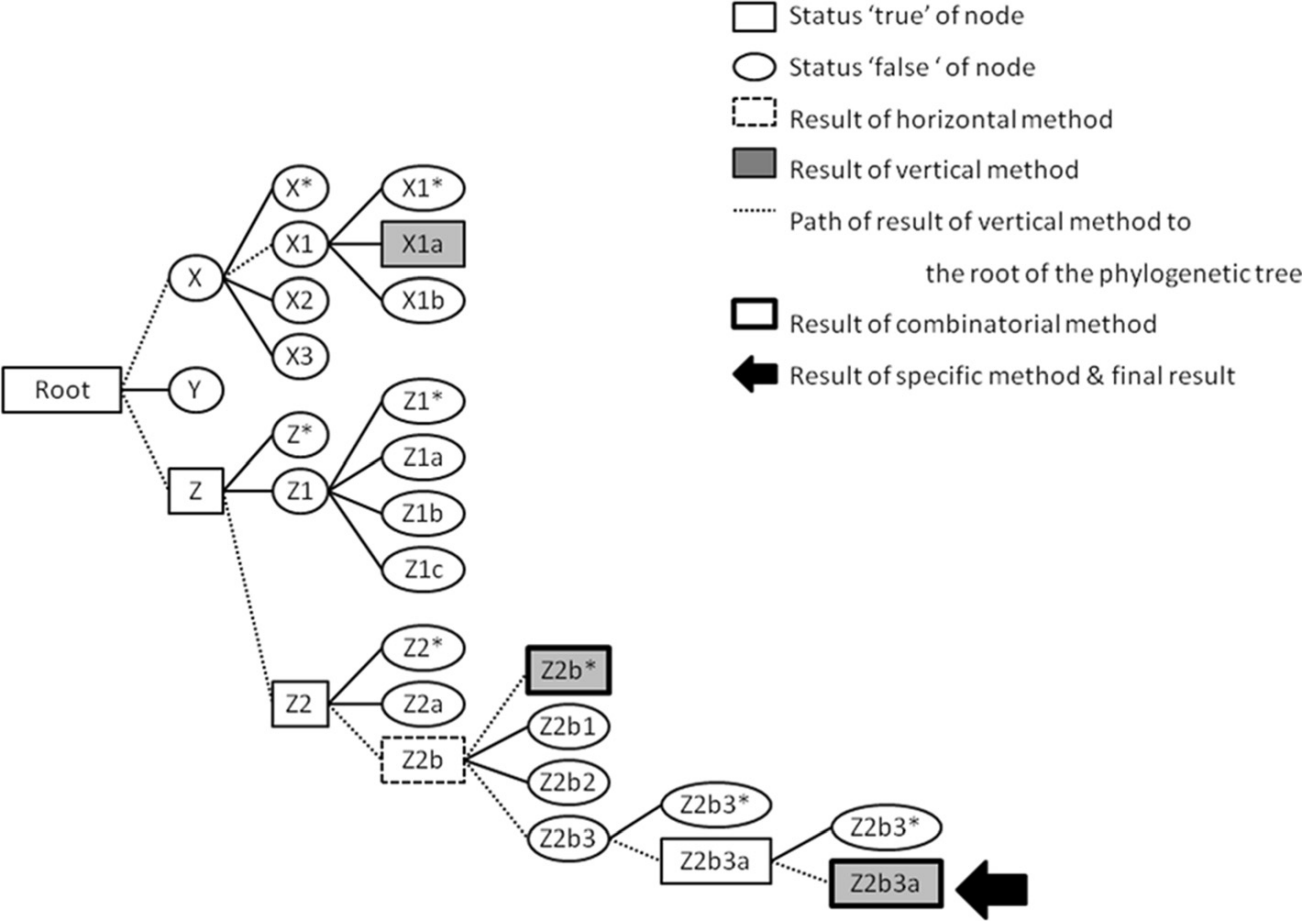
Illustrative example of the function of the AMY-tree algorithm for a fictive individual belonging to sub-haplogroup Z2b3a based on a fictive phylogenetic tree with haplogroups X, Y and Z.

The strength of the AMY-tree algorithm is that it is a combination of several strategies which use all information from the SNP calling in the analysis while also taking into account mistakes in the SNP calling and/or phylogenetic tree. A fictive example of AMY-tree’s functioning is given in Figure 2 and will be used to illustrate several steps in the algorithm, described in the following paragraphs. An even more detailed example is given in the Additional file 2.

First of all, the algorithm will determine the status of each Y-SNP in the Y chromosomal phylogenetic tree. Each Y-SNP will receive an allelic state code: 0, 1 or −1 indicating the ancestral, mutant or other allelic state respectively. These statuses depend on both the called Y-SNPs and the reference genome: if a SNP is not called, the allelic state of the reference genome is used. This could be initially wrong but in the next step the quality of the SNP calls are checked and if the quality is insufficient the allelic states of the reference genome are not taken into account for further analysis.

The first requirement to determine a (sub-)haplogroup is to check the quality of the SNP calls. There are two categories: excellent data with a ‘sufficient’ SNP calling quality and not excellent data with an ‘insufficient’ SNP calling quality. Each sample will be divided in one of these two categories based on their quality of the SNP calls. The SNP calling quality of the Y chromosome of a sample can be estimated because the reference is a composed Y chromosome of several sub-haplogroups including haplogroups G and R [12] although an individual belongs to only one sub-haplogroup. In other words, SNP calls will always be expected and based on those expectations the quality will be estimated. The quality of the Y-SNP calling is determined via the so-called ‘Call quality test’. This test will survey the allelic state of several pre-defined and well-known Y-SNPs reported in [21] and [11]. The ‘Call quality test’ with all its criteria is explained in detail in Additional file 3. The haplogroup of the sample with sufficient SNP calling quality will be quite good determined since also the data of the reference genome is taken into account. The ideal haplogroup of samples with insufficient SNP calling quality cannot be determined since none of data of the reference genome is taken into account but the determined haplogroup will be closer to the ideal one than when the reference genome is taken into account.

In the next step, the status of each node in the phylogenetic tree is determined; the determination of this Boolean status (‘true’ or ‘false’) depends on the Y-SNP calling quality. When the ‘Call quality test’ reports a sufficient Y-SNP calling quality, all statuses of Y-SNPs (both called and of reference genome) will be used to calculate the percentage mutant SNPs. For sufficient SNP calling quality samples, a percentage of more than 85% is needed to call a node ‘true’. When the ‘Call quality test’ reports an insufficient Y-SNP calling quality, all mutant alleles of the reference genome are ignored when calculating the percentage mutant SNPs and a percentage of more than 5% is needed to call a node ‘true’. In Figure 2 the ‘true’ nodes are indicated with a rectangle and the ‘false’ nodes with an oval for a specific fictive sample.

The AMY-tree search algorithm which uses the phylogenetic tree and the statuses of all nodes to determine the (sub-)haplogroup of the sample can be divided in several sub-algorithms. First, the vertical sub-algorithm checks which leaf nodes of the phylogenetic tree are ‘true’. When considering a phylogenetic tree with the root on the left site and the leaves on right site, all leaves are positioned vertically hence the name of this method. The rectangles filled in grey in the example of Figure 2 are the ‘true’ leaves and the results of the vertical method: X1a, Z2b* and Z2b3. However, as this method does not go into the tree but only checks the leaves, many false positive haplogroups can be returned with this method. Therefore we also created a horizontal sub-algorithm which goes through the tree from root to leaves via internal nodes. In the phylogenetic tree we considered, the method goes more or less horizontally from left to right hence the name of this method. This second algorithm starts at the root of the tree and checks which child nodes are ‘true’. When one or more are ‘true’, the child nodes of those nodes are also checked. It continues in this way until there are no more child nodes or until none of the child nodes is ‘true’. The last nodes that were ‘true’ are then returned. In our example, only one result is returned with this horizontal method, namely Z2b (Figure 2). Next, we combine the results of the vertical (leaf nodes) and horizontal (internal nodes) methods in order to get the best solution. Only the vertical (sub-)haplogroups in leaves which have at least one horizontal (sub-)haplogroup as ancestor in the phylogenetic tree, will be retained by the third algorithm. The consequences of this combinatorial algorithm is that most false positive vertical (sub-) haplogroups will be removed. Only Z2b* and Z2b3a are retained in our example since they have both Z2b as ancestor unlike Z1. As it is still possible, especially for the insufficient SNP calling quality samples, that leaves which share a large part of their path, are all still a result of the combinatorial algorithm e.g. Z2b* and Z2b3a in the example. In that case, a fourth algorithm is used which gets the (sub-)haplogroup that is most specific, namely Z2b3a in the example. When there are still multiple (sub-)haplogroups left after this specific algorithm, they are all returned as a result of the AMY-tree.

#### Output

The analysis file reports the ‘Call quality test’ score, the results of the vertical, horizontal, combinatorial and specific sub-algorithms and the determined sub-haplogroup of each sample. The name of the sub-haplogroups is given according to both nomenclatures proposed by [11], namely the alphanumeric nomenclature (e.g. R1b1b2a2*) and the nomenclature based on the defining SNP mutation for the sub-haplogroup (e.g. R-P312*). A warning will be given when the ‘Call-quality test’ revealed that the Y-SNP calling was insufficient. In this case, extra caution has to be taken to interpret the results due to the high occurrence of non-called SNPs and false positive SNPs. Based on the results of the sub-algorithms, it is possible for the user to find out if there was a problem on a certain phylogenetic level when the horizontal and vertical algorithms do not show the same result or when the vertical algorithm shows several possibilities.

The SNP status file shows the state of all Y-SNPs which are given in the Y-SNP mutation conversion file. In other words, for each already reported Y-SNP the user will find out if the sample has the ancestral (0), mutant (1) or other (−1) state. Moreover, it is also reported in this list if the mentioned state is obtained by calling or by derivation from the reference. The ConvNotTree file contains all Y-SNPs which have the mutant state and which are mentioned in the Y-SNP conversion file but are not present in the phylogenetic tree that was used. All synonyms of these SNPs are as well mentioned in this file. In the New SNPs file all the Y-SNPs are listed for which the phylogenetic position is not yet determined and which are not yet reported in the Y-SNP conversion file. This list is useful to detect novel yupp-SNPs on different phylogenetic levels.

### WHY conversion software

Most SNP calling programs for whole genome sequencing data such as GATK unified genotyper [26], RTG investigator, SOAPsnp [27], atlas-snp2 [28] and VarScan [29] create an output file in the variant call format (vcf). Therefore we developed a WHY conversion tool which can convert the vcf in the required tab separated values format of AMY-tree. WHY only selects the SNPs on the Y chromosome and it ignores any other variants on the Y chromosome. Besides vcf the WHY tool also can convert the Complete Genomics Analysis (cga) format. Since vcf and cga are the formats used mostly for SNP calling files, only these two are predefined in the WHY tool.

### Phylogenetic trees

The last published version of the full phylogenetic tree with all human NRY lineages was published by [11], further called as the ‘Karafet tree’. In the mean time, lots of studies have described additional Y chromosomal variation. 18 publications which described additional (sub-)haplogroups were consulted to obtain an updated tree. First, new main haplogroups were described or new SNPs were determined which changes the relationship between the main haplogroups [2,21,30,31]. Next, new sub-haplogroups or rearrangements between sub-haplogroups were described within haplogroup A [21], B [32], E [13,33], G [16,22,34-36], H [30], I [31,33], J [35], O [2,14], Q [37], R [38-40] and T [41]. Based on these publications, an updated and complete phylogenetic NRY tree (as per May 16, 2012) was constructed, further called as the ‘Updated tree’. We developed a file of the Karafet tree as well of the Updated tree which contains all full phylogenetical information needed as input for AMY-tree (Additional file 1: Table S2 and S3). It has to be noticed that there is no indel calling involved in the analyses as previously discussed and that the tree files have to be adapted to this. Therefore, 27 and 28 sub-haplogroups are not specifically defined in the Karafet tree and the Updated tree, respectively. The result of AMY-tree will always make aware of this if it is relevant for the determination of the sub-haplogroup for a certain sample. AMY-tree is created in such a way that each user can easily upload their own alternative Y chromosome tree for their analyses.

### Dataset of whole genome SNP calling profiles for testing AMY-tree

To test the AMY-tree software package, we collected 118 full genome SNP calls of in total 109 males available from four different projects (Additional file 1: Table S4). The four projects differ from each other based on the used NGS platforms and sequence coverage (high, medium and low coverage).

First, high coverage whole genome data of 35 males was collected. This data was made freely available by the commercial company ‘Complete Genomics’ (Analysis Pipeline version 2.0). The samples were sequenced with the DNA nanoball sequencing [42] with an average genome wide coverage of about 80x. They are associated with eleven different populations with their origin in Africa, America, Asia and Europe. Eight samples are known to be biological paternally related to each other and their Y chromosomes belong therefore to the same evolutionary lineage. The SNPs were called using the Complete Genomics Analysis tool which was especially designed to analyze data from Complete Genomics.

Next, one genome was available from the Irish genome project described in Tong *et al.*[12]; the sample from an autochthonous Irish male was sequenced with a 10.6× coverage on the Illumina platform and SNPs are called with the glfProgs software.

Moreover, five genome wide SNP calling profiles from whole genome and exome sequencing of samples with African origin were used. This Khoisan and Bantu genomes project was described in Schuster *et al.*[43]. For two samples we only have data from 16× coverage exome sequencing on the Roche/454 Titanium platform. Whole genome sequencing information is available for the other three sample: one sample is sequenced on the Roche/454 GS FLX platform with a 10.2× coverage and on the Roche/454 Titanium platform with 12.3× coverage (the results of the SNP calling was merged for those two analyses), another sample was sequenced using the Roche/454 Titanium platform with 2× coverage, and the last samples have been sequenced on the SOLiD platform with 30× coverage. The SNPs were called using the software Newbler (for Roche/454), Corona Lite (for SOLiD) and MAQ10 (for Illumina).

Finally, whole genome data of 77 individuals was collected from the first pilot study of the 1000 Genomes project which were sequenced on several platforms (Illumina, SOLiD and 454) with an on average 4× sequence coverage [44]. The SNP calling was performed by the GATK Unified Genotyper. These samples belong to four populations with an ancestral origin in Africa, Asia and Europe. Nine of the 77 samples were also sequenced with the Complete Genomics and were used to compare the results of both experiments.

## Results & discussions

### Quality Y-SNP allele calling

**Figure 3:**
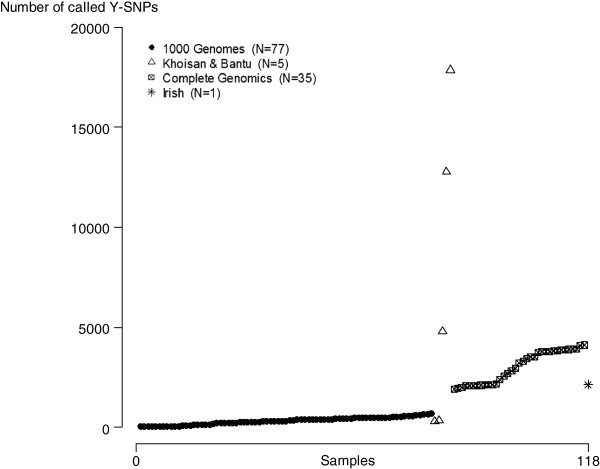
**Number of called Y-SNPs for the 118 samples of different genome sequencing projects.** All samples are ordered on the x-axis according to their project and their number of called Y-SNPs.

Sufficient Y-SNP calling quality was found for all the high sequence coverage data of Complete Genomics, the medium sequence coverage data of the Irish Genome project and two medium coverage samples of the Khoisan and Bantu genomes project. Insufficient Y-SNP calling quality was observed for all the 1000 Genomes samples which had low sequence coverage and the three other samples of the Khoisan and Bantu genomes project which had also low sequence coverage or of which only the exome was sequenced (Additional file 2: Figure S1). This was expected when the amount of reported Y-SNPs were compared between all the 118 genomes (Figure 3). However, a higher number of called Y-SNPs does not always imply a good call quality test score as the Y chromosome under study belongs to another main haplogroup which is phylogenetically highly different from the one of the reference genome e.g. one sample of the Khoisan and Bantu project with a high number of called Y-SNPs but low call quality (Additional file 2: Figure S2).

### Y chromosomal sub-haplogroup determination

**Figure 4:**
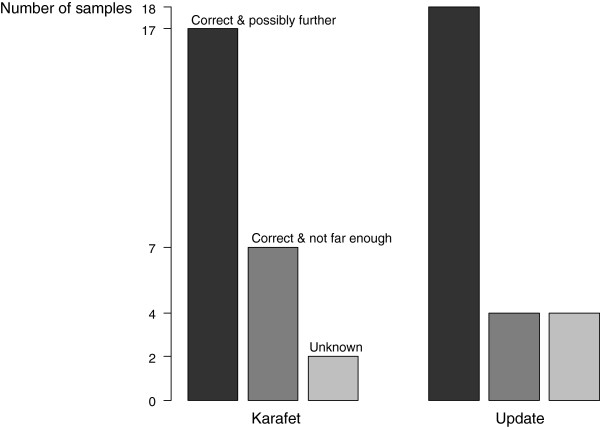
**Comparison of the results of 26 samples analysed by AMY-tree and their results obtained by previously reported SNP array genotyping.** ‘Correct & possibly further’ means that the same sub-haplogroup was determined for a sample for both approaches or that AMY-tree resolved a further phylogenetic level than by earlier SNP array analyses. ‘Correct & not far enough’ means that the same sub-haplogroup was determined for a sample for both approaches but that AMY-tree not resolved the higher observed phylogenetic level than by earlier SNP array analyses. For samples labeled with ‘Unknown’ the (sub-) haplogroup could not be determined by AMY-tree in contrast by SNP arrays.

For the samples with a sufficient Y-SNP calling quality, namely all 35 Complete Genomics samples, the Irish Genome sample, one Khoisan genome and the Bantu genome sample, 17 different Y chromosomal lineages were determined based on the Karafet tree and 18 based on the Updated tree (Additional file 1: Table S4). As expected, the eight individuals which were paternally related showed identical Y chromosomal sub-haplogroups. Next, as already reported by [12], the Irish genome sample belongs to sub-haplogroup R1b1b2a2 (R-P312*) according to the Karafet tree and to R1b1b2a1a2e (R-M529) according to the Updated tree. The determinations of the Khoisan and Bantu genome samples with sufficient SNP calling quality were also in accordance with the results given in [43]. An overview of the good accordance of the sub-haplogroups determinations based on AMY-tree and the sub-haplogroups reported earlier in the literature based on SNP array genotyping for 26 samples, is given in Figure 4 and Additional file 1: Table S5.

For the samples with an insufficient Y-SNP calling quality, namely all the sixty eight 1000 Genomes samples and three other Khoisan genome samples, only the vertical algorithm revealed results as the horizontal algorithm always stopped at the root. For four samples, the vertical algorithm did not give as well an assignment. Two Khoisan Y chromosomes were assigned to haplogroup A based on the Karafet tree but could not be assigned based on the Updated tree. This can be explained by the fact that the Updated tree uses only Y-SNPs described by [21] to define the sub-haplogroups A1a, A1b, A2 and A3. However, there are several Y-SNPs which were already described within haplogroup A by [11] but these were not included in the new [21] phylogeny. Therefore, there is no indication about the phylogenetic position of all the known Y-SNPs described earlier for haplogroup A and those are not yet included in the Updated tree. The determinations of those three Khoisan genome samples with insufficient SNP calling quality based on the Karafet tree were in accordance with the results given in [43]. Based on the Karafet tree, six individuals from the 1000 Genomes project gave two different results which does not correspond with each other (e.g. E* and I1*). Based on the Updated tree, two different results were generated for two individuals of whom one also gave two different results with the Karafet tree. The reason for the observation of several phylogenetic lineages assigned to one single individual is most likely the low sequence coverage since it can lead to more false positive SNP calls. When excluding these samples, 21 and 23 different Y chromosomal lineages were noticed for the 1000 Genomes samples; some of them are not further defined than C*, E*, O* and P* (Additional file 1: Table S4).

When comparing the results of the nine genomes which were sequenced as well by Complete Genomics as by the 1000 Genomes project, the difference between both projects is clear. Four samples give the same results for both methods based on the Karafet tree and five samples based on the Updated tree. For the other couples, it is clear that the results of the 1000 Genomes project always shows a less in-depth phylogenetic level than the results of Complete Genomics; e.g. individual NA07357 which belongs to R1* or R-M173* based on the 1000 Genomes data *versus* R1b1b2a2g or R-U152* based on the Complete Genomics data. Of course, this is the result of the much lower coverage sequencing circumstances in the 1000 Genomes project (average coverage is 4×) *versus* Complete Genomics (average coverage is 80×). Under low coverage sequencing circumstances, on average less than 5x coverage per site per individual, accurate SNP calling is indeed difficult, and there is often considerable uncertainty associated with the results. To reduce the uncertainty associated with SNP calling it is necessary to sequence deeply with at least 20x coverage [18]. This is the case for heterozygous SNPs in the autosomal chromosomes; for the haploid Y chromosome a 10× sequencing coverage could be sufficient.

**Figure 5:**
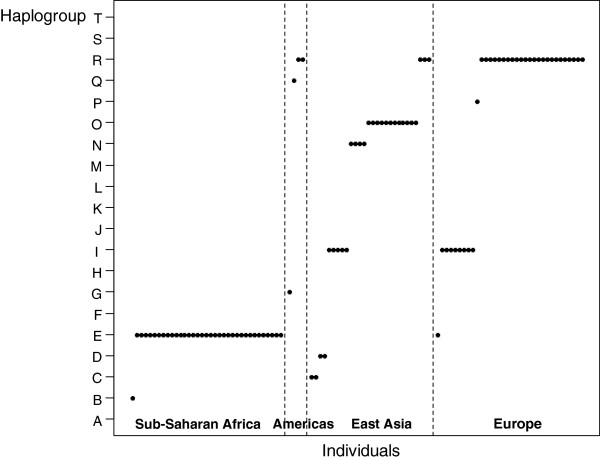
Distribution of the determined haplogroup by AMY-tree based on the update tree per geographical origin of sample.

Finally, the results of the sub-haplogroup assignments were all expected based on the ancestral origin of the sampled populations as shown in Figure 5. As expected according to [2], the Africans belongs to haplogroups A, B or E, the Europeans mainly to haplogroups R and I, and the Asians mainly to haplogroups O, D and C. However, only one from the four Americans belongs to the single major native lineage in the Americas, namely haplogroup Q defined by M242 [45]. Because of the strong admixture events between Native Americans and Europeans since 1491 [1], the other three Americans belong to typical European sub-haplogroups as the observed haplogroups R and G.

### Confirmation and ambiguities of the phylogenetic tree

Based on the 109 test samples, AMY-tree did not report any inconsistency within the Karafet and Updated tree, exclusively some small ambiguities. These results confirm the well-established backbone and the sub-haplogroup determination of the currently used Y chromosomal phylogenies. Although the low number of test samples, some issues are however found dealing with recurrent mutations and wrong reported mutation conversions. First, Y-SNP V218 was mutant for all the three Complete Genomics samples and the four 1000 Genomes samples belonging to I1* (I-M253*), in contrast to all other tested samples. Nevertheless, the mutant allele of V218 is characteristic for sub-haplogroup A2, reported for the first time by [21]. Therefore, there is evidence that V218 has a recurrent mutation within haplogroup I. This SNP is excluded from the AMY-tree analysis due to the fact that this recurrent mutation influences otherwise the results. Further research is required to reveal the phylogenetic level wherein the recurrent mutation occurred.

Next, some problems were noticed with the reported mutation conversions. The ancestral and mutant alleles are not always well reported in manuscripts or databases. After analysis, we changed the conversion of V161 and V100 (Additional file 1: Table S1). Moreover, the mutant allele was sometimes different from what was reported, namely for P321, L234 and L1015 this was the case. For those SNPs, this inconvenience was already reported on the ISOGG website.

Finally, an ambiguous result was found for Y-SNP MEH2 within haplogroup Q; this was also previously reported based on earlier SNP typing in a SNaPshot multiplex for haplogroup Q [15]. Since there was no guarantee for a correct analysis, this SNP was excluded from the analysis.

### ConvNotTree Y-SNPs

‘ConvNotTree Y-SNPs’ are interesting to detect new interesting Y-SNPs for which the mutation conversion is already known but which are not included yet in the phylogenetic framework. For some of these Y-SNPs, a suggestion for their phylogenetic position is also known on the ISOGG website (http://www.isogg.org) but not yet scientifically verified. For the Complete Genomics samples, we found a mutant allele for on average 12.8 known SNPs per sample if we take already all Y-SNPs of the Updated tree out of the analysis; haplogroup R1b samples have on average 3.1 Y-SNPs, haplogroup E samples have on average of 23.8 Y-SNPs and haplogroup I samples have on average 20.7 Y-SNPs. For the 1000 Genomes samples, we found a mutant allele on average 5.4 known SNPs per sample if we take already all Y-SNPs of the Updated tree out of the analysis; haplogroup R1 sample have on average 1.2 Y-SNPs, haplogroup NO have on average 1.3 Y-SNPs, haplogroup E have on average 12.1 Y-SNPs and haplogroup I have on average 9.1 Y-SNPs. For samples that were analysed with both platforms, the ‘ConvNotTree Y-SNPs’ which were found by 1000 Genomes were always a subset of those found by Complete Genomics.

The list of ‘ConvNotTree Y-SNPs’ was identical for all eight individuals which were paternally related. However, one individual (NA12883) revealed one extra mutant SNP, namely L374. According to ISOGG, SNP L374 should be only mutant within haplogroup G instead of in sub-haplogroup R-P312* where to all relatives belongs to. Therefore, this SNP call is a false positive or a recurrent – *de novo* mutation in this single individual and is an example that there are still ambiguities with SNP calling even for high coverage sequencing. Almost all ‘ConvNotTree Y-SNPs’ within haplogroup E samples are recently described SNPs and reported on the ISOGG website defining already existing lineages as haplogroup E1b1a (E-M2) like Z1101, Z1107, and Z1116. However, ‘ConvNotTree Y-SNPs’ could perfectly diverge the three sub-haplogroup I1* (I-M253*)-samples (with SNPs Z73, Z63 and Z140) as well as the three sub-haplogroup R1b1b2a1a2g* (R-U152*)-samples (with SNPs Z144, Z145, Z146 and Z56) from each other, as the ISOGG website already reported. Therefore, the ISOGG-tree may in addition with AMY-tree be useful to revise scientifically some (sub-)haplogroups as here for I1 (I-M253) and R1b1b2a1a2g (R-U152).

### Yupp-SNPs

Numerous Y-SNPs with unknown mutation conversion or phylogenetic position were identified in the test samples. For the Complete Genomics samples, on average 2899.6 yupp-SNPs were found. Sub-haplogroup R1b samples with on average 2066 yupp-SNPs reported, showed less yupp-SNPs than haplogroup E and I with an average of 3688 and 3301 yupp-SNPs respectively. For the 1000 Genomes samples, on average 286 yupp-SNPs were found. Haplogroup R1b samples with on average 65 yupp-SNPs reported a lower number than haplogroup E, NO and I with an average of 418, 235 and 401 yupp-SNPs respectively. The 1000 Genomes samples mentioned on average only 10% of the yupp-SNPs reported based on Complete Genomics because of the strong difference in sequence coverage. The reason that haplogroup R samples always show less yupp-SNP calls is that the reference genome is composed of multiple individuals belonging to different haplogroup R samples [12]. The number of discovered yupp-SNPs for the Irish Genome with AMY-tree, namely around 2100 is exactly the number of the reported novel Y-SNPs by [12] based on the same data. Exceptional numbers of yupp-SNPs were found for the two samples of the Khoisan and Bantu genomes project with high SNP calling quality, namely 12,515 and 17,564 SNPs (Additional file 1: Table S4). The reasons for this high numbers are the high sequence coverage with several NGS methods for these samples and the strong difference in phylogenetic position of these Y chromosomes in comparison with the reference genome.

Nevertheless, the results for the eight individuals which are paternally related with each other, revealed that there is always a chance for false positive SNP calls, also for samples sequenced with high coverage. The numbers of yupp-SNPs of these eight samples differ from each other: large numbers of yupp-SNPs are only reported in one individual or are missing in one individual. The reason is most likely false positive calls, more than *de novo* Y-SNPs, due to the low observed Y-SNP mutation rates in deep rooted pedigrees [46].

## Conclusions

The AMY-tree software was validated successfully based on 118 full genomes from 109 males with different geographical origins. The samples were analysed on several NGS platforms for whole genome sequencing with different sequence coverage depths. Therefore, AMY-tree may partly solve four issues of future Y chromosomal research:

1) It is possible to automatically determine the phylogenetical lineage to which a sample belongs to based on whole genome sequencing data without massive time-consuming efforts and therefore also avoiding laboratory and analysis errors. At this moment, SV calling is still difficult and inefficient in whole genome sequencing applications [25]. As such, indels were excluded from the analysis to maintain the required quality for AMY-tree. Therefore all end lineages defined by only (an) indel(s) may not yet be determined automatically. Further research need to focus on the inclusion of SV calling as well for Y-chromosome research.

2) Y-SNPs which are not yet reported in scientific publications but which may be crucial for some clarification of the Y chromosomal tree may now be detected efficiently. It is of course necessary to take into account that false positive SNP calls are always possible. Confirmation of those SNPs is possible by laboratory work or by future bio-informatic tools which may combine several AMY-tree results to verify it *in silico*.

3) It is possible to verify present and future Y chromosomal phylogenies, in addition to the detection of Y-SNP recurrent mutations and wrongly reported Y-SNP conversions based on whole genome SNP calling data. As long as the results are observed in at least two independent samples, it may confirm the observations.

4) The state of the art Y chromosome phylogeny will be easier to keep up to date when there will be an exponentially amount of Y chromosomal data in the future. This is possible due to the fact that data of the whole Y chromosome is collected for each individual such that the position of certain future detected SNPs will be scored as well in old sequenced samples. Moreover, the proposed tab separated value format in AMY-tree is useful to describe the up to date phylogenetic tree, especially for the massive expected Y chromosomal tree. This approach is already for a long time present for the mtDNA phylogenetic tree via http://www.phylotree.com[47].

The software AMY-Tree (inclusive the WHY-program and the user manual) is freely available on the website bio.kuleuven.be/eeb/lbeg. On this website, at least every six months (unless no new data has appeared), an update will be given of the SNP conversion file and of the Updated Y chromosomal phylogenetic tree in tab separated value format based on scientific publications.

## Competing interests

The authors declare no conflict of interest.

## Authors’ contributions

Research design & supervision: MHDL; Programming: AVG; Writing: AVG & MHDL; Commenting on manuscript: AVG, RD & MHDL. All authors read and approved the final manuscript.

## Supplementary Material

Additional file 1: Table S1 The latest version of the Y-SNP conversion file (May 2012). This list contains the name, the synonyms, the RefSNP ID, the position on the Y-chromosome according to references NCBI36 (Hg18) and GRCh37 (Hg 19) and the mutant conversion state (Ancestral allele -> Mutant allele) of all reported Y-SNPs. **Table S2.** Table-format of the latest full published Y-chromosomal phylogenetic tree of [11]. For each reported Y-chromosomal (sub-)haplogroup, the phylogenetic lineage whereto the (sub-)haplogroup belongs, called the ‘parental lineage’, and the defining Y-SNPs of the lineage are given. **Table S3.** Table format of the updated Y-chromosomal phylogenetic tree based on the most recent scientific publications (May 2012). For each reported Y-chromosomal (sub-)haplogroup, the phylogenetic lineage whereto the (sub-)haplogroup belongs, called the ‘parental lineage’, and the defining Y-SNPs of the lineage are given. **Table S4.** Characteristics and AMY-tree results of the test panel with samples from the Complete Genomics (CG), Irish genome (IrG), Khoisan and Bantu Genomes (KBG) and 1000 Genomes (1000G) projects. For each sample the population as well as the ancestral continent of the population is given, next to the relationship with other samples of the test panel. Abbreviations of the populations are ASW: African ancestry in Southwest USA; CEU: Utah residents with Northern & Western European ancestry from the CEPH collection; CHB: Han Chinese in Beijing, China; GIH: Gujarati Indian in Houston, Texas, USA; JPT: Japanese in Tokyo, Japan; LWK: Luhya in Webuye, Kenya; MKK: Maasai in Kinyawa, Kenya; MXL: Mexican ancestry in Los Angeles, California, USA; NK: Khoisan from Northern Kalahari; SA: Bantu from South Africa; SK: Khoisan from Southern Kalahari; TSI: Toscans in Italy; PUR: Puerto Rican in Puerto Rico; YRI: Yoruba in Ibadan, Nigeria. **Table S5.** Characteristics and AMY-tree results of samples of which the sub-haplogroups was previously reported in literature. Samples are taken from the Complete Genomics (CG), Irish genome (IrG), Khoisan and Bantu Genomes (KBG) and 1000 Genomes (1000G) project. For each sample the population as well as the ancestral continent of the population is given, next to the relationship with other samples of the test panel. Abbreviations of the populations are CEU: Utah residents with Northern & Western European ancestry from the CEPH collection; MXL: Mexican ancestry in Los Angeles, California, USA; NK: Khoisan from Northern Kalahari; SA: Bantu from South Africa; SK: Khoisan from Southern Kalahari; TSI: Toscans in Italy; PUR: Puerto Rican in Puerto Rico; YRI: Yoruba in Ibadan, Nigeria. The sub-haplogroup as it is given in the literature is compared to the determined sub-haplogroups in Karafet and Update tree. Click here for file

Additional file 2: Figure S1 Call quality test scores for 118 samples from different genome sequencing projects created within the AMY-tree algorithm. All samples are ordered according to their project and their number of called Y-SNPs. **Figure S2.** Relationship between call quality test score and number of Y-SNPs called against hg18 for 118 samples from different genome sequencing projects. Click here for file

Additional file 3 Supplementary Method. Click here for file
