# Supplementary material for: AMY-tree: an algorithm to use whole genome SNP calling for Y chromosomal phylogenetic applications
Source: BMC Genomics. 2013 Feb 13;14:101. doi: 10.1186/1471-2164-14-101 (PMC3583733; doi:10.1186/1471-2164-14-101)
Supplement: Additional file 2: Figure S1 — Call quality test scores for 118 samples from different genome sequencing projects created within the AMY-tree algorithm. All samples are ordered according to their project and their number of called Y-SNPs. Figure S2. Relationship between call quality test score and number of Y-SNPs called against hg18 for 118 samples from different genome sequencing projects. [file 1471-2164-14-101-S2.docx]

**Supplementary Figures**

**Figure S1** Call quality test scores for 118 samples from different genome sequencing projects created within the AMY-tree algorithm. All samples are ordered according to their project and their number of called Y-SNPs.

**Figure S2** Relationship between call quality test score and number of Y-SNPs called against hg18 for 118 samples from different genome sequencing projects.
