# Supplementary material for: AMY-tree: an algorithm to use whole genome SNP calling for Y chromosomal phylogenetic applications
Source: BMC Genomics. 2013 Feb 13;14:101. doi: 10.1186/1471-2164-14-101 (PMC3583733; doi:10.1186/1471-2164-14-101)
Supplement: Additional file 3 — Supplementary Method. [file 1471-2164-14-101-S3.docx]

**Supplementary Method**

**Algorithm**

*1. Status of nodes*

This is a fictive but realistic example of a Y-chromosomal phylogenetic tree. Nodes which names end with an asterix are the paragroups, as it is given in the latest published official Y-chromosomal tree of Karafet et al. (2008). The first step of the AMY-tree algorithm is to determine the status of each node for a given sample. All nodes which have a mutant allele state are black, the ones with an ancestral state are white.

*2. Horizontal method*

Next, the horizontal method starts at the root and goes to each mutant child node until the mutant child nodes are leaves or until there are no more mutant child nodes. The result of the horizontal method is in this example Z2. The path to get to this horizontal result is indicated with a dashed line.

*3. Vertical method*

Thereafter, all the mutant leaves of the phylogenetic tree are selected as results of the vertical method. In this examples X1a, Z2*, Z2b3* and Z2b3a1 are the results of the vertical method.

*4. Combinatorial method*

Horizontal and vertical results are combined to remove false positive results. Only the vertical results which have a horizontal result in their path from leaf to root are kept. The vertical result X1a will be eliminated in this example.

*5. Specific method*

If there are still multiple combinatorial results, like in this example, a specific method is applied to get the most specific result as final result. This is done by keeping the result which shows the most overlap of his path with the paths of the other results and which has a deeper phylogenetic level. As Z2b3a1 has the most overlap of his path with that of Z2b3* (from root to Z2b3), this one is the final haplogroup of the fictive sample.

**Call quality test**

This test will determine the quality of the called SNPs of the sample which corresponds to the influence the reference genome would have on the result of AMY-tree as it is assumed that an individual may only belong to one single haplogroup and that the Y-chromosome of the reference genome is composed from multiple individuals belonging to several haplogroups. The ‘Call quality test’ will subdivide each sample to one of two categories, namely low and high Y‑SNP calling quality.

First, the test will determine to which well-defined haplogroup (A1b, A1a, A2, A3, B, C, DE or F) the sample belongs to based on the Y-SNPs reported in Cruciani et al. (2011) and Karafet et al. (2008):

A1b = V148, V149, V150, V151, V153, V154, V157, V158, V159, V161, V162, V163, V164, V165, V166, V167, V169, V172, V173, V176, V177, V181, V190, V196, V223, V225, V229, V233, V239.

A1a = V4, V14, V15, V25, V26, V28, V30, V40, V48, V53, V57, V58, V63, V76, V191, V201, V204, V214, V215, V236.

A2 = V50, V61, V70, V72, V79, V80, V81, V82, V180, V188, V192, V198, V200, V224, V228, V242.

A3 = V1, V10, V51, V56, V66, V67, V89, V98, V155, V156, V160, V193, V194, V230, V243.

B = V62, V75, V78, V83, V85, V90, V93, V94, V185, V197, V217, V220, V227, V234, V237, V244.

C = V20, V77, V86, V182, V183, V184, V199, V219, V222, V232, RPS4Y711, M216, P184, P255, P260.

DE = M145, M203, P144, P153, P165, P167, P183.

F = P14, M89, M213, P134, P135, P136, P138, P139, P140, P142, P145, P148, P149, P151, P158, P159, P160, P163, P166.

After determining the haplogroup based on the highest score (= highest percentage mutant SNPs), the ‘Call quality test’ controls how many SNPs of this haplogroup for the sample is indeed mutant and how many SNPs of the other haplogroups are indeed ancestral for the sample. When the allelic states are less than 90% correct, the SNP calling quality of the sample is ‘low’. When the allelic states are more than 90% correct, the SNP calling quality is expected to be ‘high’. However, an extra test is required if the determined haplogroup of the sample is F. The percentage of 90% as criteria is defined after numerous test-runs with simulated samples of different SNP call qualities.

For samples assigned to haplogroup F, an extra test is required whereby the sample has to be assigned to one of the three groups:

G with: M201=1, P257=1, U2=1, U3=1, U6=1, U7=1, U12=1, P231=0, P233=0, P234=0, P236=0, P238=0, P242=0, P286=0, P294=0, P225=0, P245=0.

R1 with: M201=0, P257=0, U2=0, U3=0, U6=0, U7=0, U12=0, P231=1, P233=1, P234=1, P236=1, P238=1, P242=1, P286=1, P294=1, P225=1, P245=1.

Other with: M201=0, P257=0, U2=0, U3=0, U6=0, U7=0, U12=0,P231=0, P233=0, P234=0, P236=0, P238=0, P242=0, P286=0, P294=0, P225=0, P245=0.

In these three groups, ancestral alleles are represented by ‘0’ and mutant alleles by ‘1’.

In this extra test, the sample is assigned to the group with the highest resemblance. After that, the number of mistakes between the assigned group and the real SNP call data is calculated. When the allelic states of less than 90% of the SNPs is incorrect, the SNP call quality is ‘insufficient’, otherwise it is checked if all R1 specific SNPs (M201, P257, U2, U3, U6, U7, U12) are mutant. If they are not all mutant the quality is ‘insufficient’, otherwise all R1a1a SNPs (M198, M417, M512, M514, M515, Page7) are checked. If they are all mutant the quality is again ‘insufficient’, otherwise the quality is ‘sufficient’.

**References**

Cruciani F, Trombetta B, Massaia A, Destro-Bisol G, Sellitto D, Scozzari R. 2011. A revised root for the human Y chromosomal phylogenetic tree: The origin of patrilineal diversity in Africa. American Journal of Human Genetics 88(6):814-818.

Karafet TM, Mendez FL, Meilerman MB, Underhill PA, Zegura SL, Hammer MF. 2008. New binary polymorphisms reshape and increase resolution of the human Y chromosomal haplogroup tree. Genome Research 18(5):830-838.
